# Classifications of anterior segment structure of congenital corneal opacity in infants and toddlers by ultrasound biomicroscopy and slit-lamp microscopic photographs: an observational study

**DOI:** 10.1186/s12886-024-03286-z

**Published:** 2024-01-23

**Authors:** Jing Hong, Zijun Xie, Xin Wang, Ting Yu, Siyi Ma, Hanzhi Ben, Shao-feng Gu

**Affiliations:** 1 Department of Ophthalmology, Peking University Third Hospital, Beijing, China; 2 Beijing Key Laboratory of Restoration of Damaged Ocular Nerve, Peking University Third Hospital, Beijing, China

**Keywords:** Corneal opacity, Cornea diseases, Congenital abnormalities, Ultrasound Biomicroscopy

## Abstract

**Background:**

The structural features have an impact on the surgical prognosis for congenital corneal opacity (CCO). The structural classification system of CCO, however, is lacking. Based on data from ultrasound biomicroscopy (UBM) findings in infants and toddlers with CCO, this research proposed a classification system for the anterior segment structure severity.

**Methods:**

Medical records, preoperative UBM images and slit-lamp photographs of infants and toddlers diagnosed with CCO at University Third Hospital between December 2018 and June 2022 were reviewed. According to the anterior segment structural features observed in UBM images, eyes were classified as follows: U1, opaque cornea only; U2, central anterior synechia; U3, peripheral anterior synechia combined with angle closure; and U4, aniridia or lens anomaly. The opacity appearance and corneal vascularization density observed in slit-lamp photographs were assigned grades according to previous studies. The extent of vascularization was also recorded. The corresponding intraocular anomaly classifications and ocular surface lesion severity were analysed.

**Results:**

Among 81 eyes (65 patients), 41 (50.6%) were right eyes, and 40 (49.4%) were left eyes. The median age at examination was 6.91 months (*n* = 81, 1.00, 34.00). Two (2.5%) of the 81 eyes were classified as U1, 20 (24.7%) as U2, 22 (27.2%) as U3a, 11 (13.6%) as U3b and 26 (32.1%) as U4. Bilateral CCO eyes had more severe UBM classifications (*P* = 0.019), more severe dysgenesis (*P* = 0.012) and a larger angle closure (*P* = 0.009). Eyes with more severe UBM classifications had higher opacity grades (*P* = 0.003) and vascularization grades (*P* = 0.014) and a larger vascularization extent (*P* = 0.001). Eyes with dysgenesis had higher haze grades (*P* = 0.012) and more severe vascularization (*P* = 0.003 for density; *P* = 0.008 for extent), while the angle closure range was related to haze grade (*P* = 0.013) and vascularization extent (*P* = 0.003).

**Conclusions:**

This classification method based on UBM and slit-lamp photography findings in the eyes of CCO infants and toddlers can truly reflect the degree of abnormality of the ocular surface and anterior segment and is correlated with the severity of ocular surface anomalies. This method might provide meaningful guidance for surgical procedure design and prognostic determinations for keratoplasty in CCO eyes.

## Backgrounds

Congenital corneal opacity (CCO) is a rare disease that presents in 2.2 ~ 3.11/100,000 newborns [1, 2]. Although infrequent, the opacity may cause visual deprivation amblyopia and even lifelong blindness, so keratoplasty is needed early in life [2, 3].

Keratoplasties in children are considered more complicated than those in adults as these procedures are characterized by more formidable surgery and a higher rate of graft failure in children than in adults [3]. Compared with adult eyes, paediatric eyes have lower scleral rigidity and a more crowded anterior chamber [3]. Moreover, the pathogenesis of CCO, including dermoid, sclerocornea, and anterior segment developmental abnormalities, varies and can increase the difficulty of the operation and risk of complications during and after the procedure [4]. 

The graft survival rates of paediatric penetrating keratoplasties vary widely due to distinct indications [5]. In congenital conditions, eyes with Peters anomaly, sclerocornea, and congenital glaucoma have a higher risk of graft failure [6, 7]. However, the diagnosis of CCO is still unclear thus far. The “S.T.U.M.P.E.D.” method of clinical classification takes both the signs (sclerocornea, ulcer, and Peters anomaly) and diagnosis (trauma, metabolic disorders, endothelial dystrophy, and dermoids) into consideration [4]. Although helpful for clinicians to remember the diagnoses that may cause congenital opacity, it does not provide prognostic information. To clarify the diagnostic system, a classification based on phenotype-genotype correlation has been proposed. However, the new classification is clinically impracticable for economic reasons and is unsuitable given the results of contradictory genotypes in identical phenotypes in genetic studies [4, 8, 9].

Superficial and intraocular structural features are related to the surgical prognosis regardless of diagnosis. Preoperative corneal vascularization increases the risk of graft rejection [6, 10–12]. Anterior synechia in CCO is a significant risk factor for postoperative anterior synechia, which is common in young patients and may induce secondary glaucoma and graft rejection [13, 14]. Quadrants of angle closure are associated with postoperative glaucoma [15]. Eyes with anterior segment developmental abnormalities have more preoperative complications due to trabecular meshwork dysfunction and other anterior segment structures [16–18]. Therefore, it is very important to evaluate the structural risk factors before surgery.

However, optical examination is not suitable for the observation of intraocular structure in completely opaque eyes. Therefore, ultrasound biomicroscopy (UBM) is the only non-invasive choice for high-solution anterior segment imaging for these eyes [19]. Compared with optical examinations, UBM can probe through opaque tissue, making it valuable for the accurate diagnosis of CCO eyes, the preoperative evaluation, and management decisions [19, 20]. Slit-lamp microscopy is essential for ophthalmologic examination and is the most convenient non-invasive method for ocular surface observation.

Previous studies on paediatric keratopathy showed an association between CCO severity and surgical prognosis [5]. However, a classification system for severity evaluation of preoperative structural features of paediatric eyes with CCO based on large-sample research is lacking due to the relatively low prevalence rate and poor examination compliance. In this study, the intraocular and ocular surface features of infants and toddlers with CCO were observed, classified, and analysed accordingly, with the goal of finding correspondence between the severity of the two dimensions of observation.

## Methods

### Patients

The UBM classification criteria were used for patients under three years of age who underwent preoperative UBM examination and received primary penetrating keratoplasty between December 2018 and June 2022 in Peking University Third Hospital. Patients with a history of surgery that changed the anterior segment structure were excluded from this study.

The basic information and medical histories of all patients were reviewed. The collected data included the patient’s sex, date of birth, and surgical history. Reports of preoperative UBM examinations and images of the ocular surface captured by slit-lamp microscopy were studied.

### UBM examination

UBM examinations were performed using SW-3200 L UBM 50 MHz (SUOER, Tianjin, China) by the same experienced technician. To ensure cooperation, all patients were examined under anaesthesia using oral chloral hydrate (0.3 ~ 0.5 ml/kg) sedation. First, oxybuprocaine hydrochloride (Benoxil 0.4% solution) was used for surface anaesthesia in a supine position. A contact lens cup was placed in the conjunctival sac and filled with multipurpose contact lens solution.

To observe the structure of the anterior chamber, the technician dipped a probe in water, took an axial scan to see the position and morphology of the lens, and then scanned along the limbus radially. Tangent scanning was also performed if necessary. Levels of anterior chamber angle closure and iridocorneal adhesions were observed thoroughly. Images of axial scanning, radial scanning (images of 3, 6, 9, and 12 o’clock), and particular anomalies were frozen and recorded. Finally, ofloxacin eye drops were used on the examined eyes.

### Features of UBM images

CCO patients who cooperated with oral chloral hydrate sedation were included, and only those with all UBM images for the eyes (i.e., at least one axial scan and the four images of the four-point position) were included in this study.

**Fig. 1:**
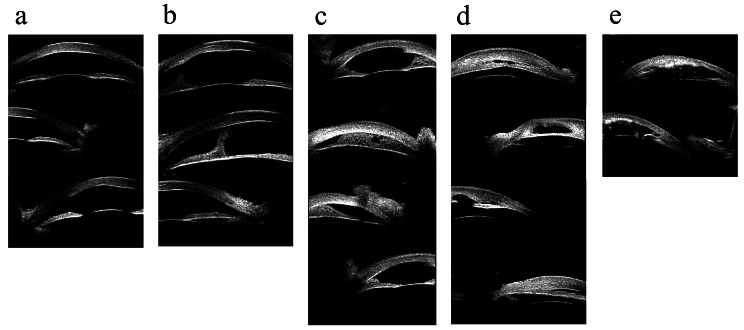
Ultrasound biomicroscopic images of different ultrasound biomicroscopic classifications. (a) Only an opaque cornea was shown in ultrasound biomicroscopic images. (b) Ultrasound biomicroscopic images showed opaque cornea and central anterior synechia. (c) Ultrasound biomicroscopic images showed an opaque cornea and peripheral iridocorneal synechia, combined with angle closure ranging less than or equal to 180 degrees. (d) Ultrasound biomicroscopic images showed an opaque cornea and peripheral iridocorneal synechia, combined with angle closure greater than 180 degrees. (e) The eyes classified as U4 are coupled with or without angle closure, and ultrasound biomicroscopic images revealed an opaque cornea with iris and lens anomaly

Based on the features of the anterior segment structure found in the UBM examination, the eyes were classified into four groups by 2 experienced clinicians independently (see Table 1; Fig. 1). Eyes classified as U4 were considered to have dysgenesis of the anterior segment.

**Table 1:** Ultrasound biomedical classification criteria

Classifications		Corneal opacity	Iridocorneal synechia	Angle closure	Lens or iris anomaly	Examples
U1		√	-	-	-	Figure 1 - A
U2		√	Central	-	-	Figure 1 - B
U3	U3a	√	Peripheral	$\leqslant$180 degrees	-	Figure 1 - C
	U3b	√	Peripheral	>180 degrees	-	Figure 1 - D
U4		√	√/-	√/-	UBM findings showed iris hypoplasia or lens anomaly, including lens dislocation, shape anomaly or aphakia.	Figure 1 - E

However, eyes classified as U4 also had anterior synechia combined with angle closure. Therefore, the ranges of angle closure were observed and recorded to distinguish the influence between anterior segment developmental abnormalities and the severity of angle closure. Closure of an anterior angle was identified if the iris covered the scleral spur in a UBM image. The closure range of an eye was classified as 0 degrees, less than or equal to 180 degrees, and more than 180 degrees.

### Features of ocular surface findings

The severity of corneal opacification and vascularization was studied based on features of the preoperative slit-lamp microscopic photographs.

**Fig. 2:**
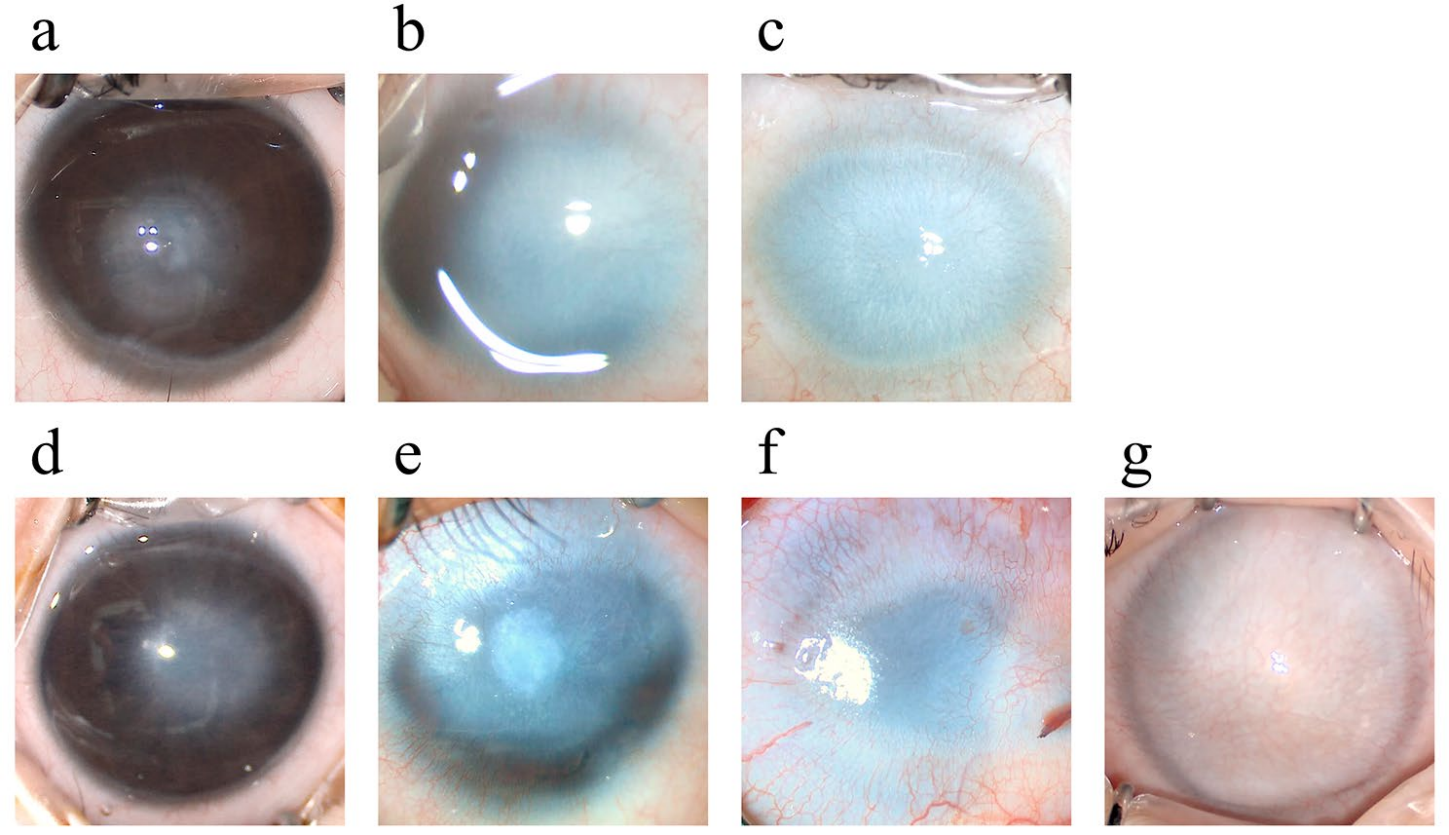
Images of different grades of ocular surface features. (a) Mild corneal haze (F1): The opacity was smaller than 1/4 of the cornea, and iris details could be seen through it. (b) Moderate corneal haze (F2): The opacity was larger than 1/4 of the cornea with the lens and iris details blocked. (c) Severe corneal haze (F3): The lens and iris were completely hidden behind the opacity. (d) Nonvascularized (V0): Opacified cornea with no sign of vascularization. (e) Mildly vascularized (V1): Straight loop-shaped fine vessels grew on the corneal limbus from 4 to 12 o’clock. (f) Moderately vascularized (V2): Two large arborizing vessels grew across the limbus at 2 and 5 o’clock. (g) Severely vascularized (V3): The opacity was completely covered by retiform vessels

Opacities were graded from 1 to 3 for severity and extent based on a modified Fantes haze scale (see Table 2; Fig. 2) [21]. The severity of corneal vascularization was classified by range involved and vessel density. The range involved was defined as 0 ~ 12 according to the clock number of corneoscleral limbus crossed by vessels. Vessel density was classified as V0 ~ V3 according to the density and morphology of vascularization (see Table 2; Fig. 2).

**Table 2:** Criteria for ocular surface feature grades

Classifications		Criteria	Examples
Modified Fantes haze grade	F1 (mild)	Opacity less than 1/4 of the cornea without interference with visibility of iris details and no vascularization.	Figure 2 - A
	F2 (moderate)	Opacity larger than 1/4 of the cornea with mild interference with visibility of iris details with or without vascularization.	Figure 2 - B
	F3 (severe)	Opacity totally hides the iris and lens with or without vascularization.	Figure 2 - C
Density of vascularization	V0 (nonvascularized)	No vascularization.	Figure 2 - D
	V1 (mildly vascularized)	Fine vessels growing as straight loops on the limbus.	Figure 2 - E
	V2 (moderately vascularized)	Large blood vessels with arborizing appearance growing from limbus to center	Figure 2 - F
	V3 (severely vascularized)	Multiple apparent vessels covering the complete corneal opacity.	Figure 2 - G

### Statistical methods

IBM SPSS Statistics for Windows, version 27.0 (IBM Corp., Armonk, N.Y., USA) was used for data analysis. Quantitative variables are described as the mean ± SD or median (n, maximum, minimum) if nonnormal, while categorical variables are shown as numbers and percentages. The Kolmogorov‒Smirnov test was used to define the data distribution. The Mann‒Whitney U test was used to analyse ranked and nonnormally distributed data, while the t test was used for normally distributed data. For comparisons between categorical data, the chi-square test was used. *P* < 0.05 was considered statistically significant.

## Results

A total of 81 eyes from 65 patients were included in our final analysis. There were 29 (44.6%) males and 36 (55.4%) females. Forty-one (50.6%) were right eyes, and 40 (49.4%) were left eyes. Thirty-six (36/65, 55.4%) of the patients had bilateral CCO, and eight (8/36, 22.2%) of them had concomitant systemic diseases, including congenital heart disease (5/8, 62.5%), urinary system disease (1/8, 12.5%), digestive system disease (1/8, 12.5%) and cleft lip (1/8, 12.5%). Fifty-two eyes of the 36 bilateral CCO patients were included. The patients’ median age at UBM examination was 6.91 (*n* = 81, 1.00, 34.00) months. One patient had undergone bilateral cyclodiode photocoagulation before the examination.

We reviewed 450 UBM images and 979 slit-lamp microscopic images of the 81 eyes. Based on the intraocular structural features of all eyes of the patients enrolled, two (2/81, 2.5%) were classified as U1 (simple corneal opacity), 20 (20/81, 24.7%) as U2 (corneal opacity with central anterior synechia), 22 (22/81, 27.2%) as U3a (corneal opacity with peripheral anterior synechia, range of angle closure less than or equal to 180 degrees), 11 (11/81, 13.6%) as U3b (corneal opacity with peripheral anterior synechia, range of angle closure larger than 180 degrees) and 26 (26/81, 32.1%) as U4 (anterior segment developmental abnormality). In the U4 group, all eyes had iris hypoplasia, and 16 (16/26, 61.5%) had an abnormal lens. Regarding the range of anterior chamber angle closure, two UBM reports classified as U4 showed no clear angle structure, and the other 79 eyes were included. Twenty-five eyes (25/79, 31.6%) had no angle closure, 29 eyes (29/79, 36.7%) had angle closure less than or equal to 180 degrees, and 25 (25/79, 31.6%) had angle closure greater than 180 degrees. Based on superficial photographs, five opacities (5/81, 6.2%) were F1 (mild), 45 (45/81, 55.6%) were F2 (moderate), and 31 (31/81, 38.3%) were F3 (severe). The slit-lamp microscopic photographs of one patient failed to show the details of neovessels and were excluded. Among the other 80 eyes, four (4/80, 5.0%) were nonvascularized (V0), 31 (31/80, 38.8%) were mildly vascularized, 19 (19/80, 23.8%) were moderately vascularized, and 26 (26/81, 32.5%) were severely vascularized. The median vascularization extent was 10.0 (*n* = 80, 0.0, 12.0) clock hours.

Compared with unilaterally involved eyes, those with bilateral CCO had more severe intraocular anomalies. There were 20 eyes (20/52, 38.4%) with bilateral CCO combined with angle closure and 22 eyes (22/52, 42.3%) with anterior segment developmental abnormalities, while ten eyes (10/29, 34.5%) had unilateral CCO combined with central anterior synechia, and 13 eyes (13/29, 44.8%) had angle closure (*P* = 0.019). Regarding the combination of anterior segment developmental abnormalities, 22 (22/52, 42.3%) bilaterally involved eyes and 4 (4/29, 13.8%) unilaterally involved eyes were noted (*P* = 0.012). In addition, bilaterally involved eyes had a larger range of angle closure (*P* = 0.009). However, the association between bilateral involvement and haze grades (*P* = 0.393), vascularization classifications (*P* = 0.091), and vascularization extent (*p* = 0.525) were found to have no statistical significance.

Further statistical tests showed a significant correlation between the severity of intraocular and superficial abnormal findings (Table 3). One U1 (1/2, 50.0%), three U2 (3/20, 15.0%), and one U4 (1/26, 3.8%) eyes had mild appearance (F1); four U2 (4/20, 20.0%), five U3a (5/22, 22.7%), six U3b (6/11, 54.5%) and 16 U4 eyes (16/26, 61.5%) had severe appearance (F3); and the other eyes were moderately opaque (F2) (Table 3, *P* = 0.003). Eye with dysgenesis (U4) showed a higher haze grade. Sixteen eyes (16/25, 61.5%) were severely opaque (F3), nine eyes (9/26, 34.6%) were moderately opaque (F2), and only one eye (1/26, 3.4%) was mildly opaque (F1) (*P* = 0.012). Regarding angle closure, four eyes (4/25, 16.0%) with no angle closure and one eye (1/29, 3.4%) with angle closure smaller than 180 degrees showed mild opacities (F1). Sixteen eyes (16/25, 64.0%) with no angle, 18 eyes (18/29, 62.1%) with angle closure smaller than 180 degrees, and ten eyes (10/25, 40.0%) with angle closure larger than 180 degrees showed moderate opacities (F2), and other eyes showed severe opacities (F3) (Table 3, *P* = 0.013).

UBM classifications showed a significant association with vascularization density. All U1 eyes and 2 U2 eyes (2/20, 10.0%) had no appearance of neovascularization (V0); 11 U2 eyes (11/20, 55.0%) and 14 U3 eyes (14/33, 42.4%) were mildly vascularized (V1); and 13 U4 eyes (13/26, 50.0%) were severely vascularized (Table 3, *P* = 0.014). Vascularization in eyes with dysgenesis was more severe (Table 3, *P* = 0.047). The median vascularization extent significantly differed among the eyes of distinct UBM classifications (Table 3, *P* = 0.001). The median extent in eyes with dysgenesis was 11.5 (26, 3.0, 12.0) clock hours, which was higher than that in normal eyes (8.5 (54, 0.0, 12.0) clock hours, *P* = 0.008). However, the range of angle closure was not related to classifications of neovascularization (Table 3, *P* = 0.081), but eyes with a larger extent of angle closure showed a larger range of vascularization (Table 3, *P* = 0.003).

**Table 3:** Correlation between superficial and intraocular findings

Classifications of UBM findings	Modified Fantes haze grades (*n* = 81)			*P* value	Vascularization density (*n* = 80)				*P* value	Median vascularization extent (*n* = 80)	*P* value
	F1 (*n* = 5)	F2 (*n* = 45)	F3 (*n* = 31)		V0 (*n* = 4)	V1 (*n* = 31)	V2 (*n* = 19)	V3 (*n* = 26)			
U1 (*n* = 2)	1 (50.0%)	1 0(50.0%)	000 (0.0%)	**0.003**	2 (100.0%)	000 (0.0%)	00 (0.0%)	000 (0.0%)	**0.014**	0.00 (2, 00.0, 0.0)	**0.001**
U2 (*n* = 20)	3 (15.0%)	13 (65.0%)	40 (20.0%)		20 (10.0%)	11 (55.0%)	3 (15.0%)	40 (20.0%)		05.5 (20, 12.0, 0.0)	
U3a (*n* = 22)	0 0(0.0%)	17 (77.3%)	50 (22.7%)		0 00(0.0%)	10 (47.6%)	6 (28.6%)	50 (23.8%)		10.0 (21, 12.0, 3.0)	
U3b (*n* = 11)	0 0(0.0%)	50 (45.5%)	60 (54.5%)		0 00(0.0%)	40 (36.4%)	3 (27.3%)	40 (36.4%)		11.0 (11, 12.0, 6.0)	
U4 (*n* = 26)	1 0(3.8%)	90 (34.6%)	16 (61.5%)		0 00(0.0%)	60 (23.1%)	7 (26.9%)	13 (50.0%)		11.5 (26, 12.0, 3.0)	
0 degree (*n* = 25)	4 (16.0%)	16 (64.0%)	50 (20.0%)	**0.013**	40 (16.0%)	12 (48.0%)	4 (16.0%)	50 (20.0%)	0.081	06.0 (25, 12.0, 0.0)	**0.003**
≤ 180 degrees (*n* = 29)	1 0(3.4%)	18 (62.1%)	10 (34.5%)		0 00(0.0%)	12 (42.9%)	7 (25.0%)	90 (32.1%)		10.0 (28, 12.0, 3.0)	
≤180 degrees (*n* = 25)	0 0(0.0%)	10 (40.0%)	15 (60.0%)		0 00(0.0%)	70 (28.0%)	7 (28.0%)	11 (44.0%)		11.0 (25, 12.0, 3.0)	

## Discussion

With the use of UBM, anterior segment structures could be determined clearly despite the homogeneous appearances of eyes with opaque congenital keratopathy, which was helpful for surgical planning and prognosis estimation [5, 22, 23]. In this study, we proposed a classification method for evaluating lesion severity based on UBM findings in infants and toddlers with CCO eyes and found it highly relevant to evaluating the severity of ocular face lesions.

Our classification system took anterior synechia, angle closure, and anterior segment developmental abnormality into consideration, which are important risk factors for graft failure in CCO eyes [13–18]. Eyes with anterior segment developmental abnormalities according to our classification system were defined as eyes with iris hypoplasia or lens anomalies in size and shape, position or transparency, indicating the contribution of gene mutations in these eyes [17]. Regarding the severity of superficial lesions, gradings of corneal scars after wound healing and opacities in congenital glaucoma eyes were referred to on the assumption that with higher scar grade came poorer vision acuity [21, 24]. Preoperative corneal vascularization is a crucial risk factor that may induce immunological rejection [6, 10–12]. Because there are few research studies in the field of congenital vascularization, our classification of corneal vascularization referred to that of acquired conditions [25]. Based on the considerations above, we developed an opacity grading and a vascularization classification for the evaluation of ocular surface lesions.

Our results showed that eyes with more severe UBM classifications (*P* = 0.003), anterior segment developmental abnormalities (*P* = 0.012), and a larger range of angle closure (*P* = 0.013) had a more severe appearance of opacities. One possible reason is that these eyes had an absence of intact corneal endothelium due to abnormal migration and differentiation of ocular neural crest cells [26]. Three neural crest migration waves were reported in anterior segment development, which started in the seventh week of gestation, and the cells eventually differentiated into corneal endothelial cells, trabecular meshwork epithelial cells, and iris stroma cells [27, 28]. The abnormal progression of this process may lead to corneal endothelium anomalies and anterior synechia [26]. Corneal endothelial cells are permeable to nutrients and waste products regulated by incomplete zonula occludens and assist in corneal hydration maintained by the pump system to guarantee optical clarity [29]. Destruction of the integrity of the corneal endothelium may cause corneal stromal edema, thus reducing optical transparency. Moreover, lens vesicles were formed two weeks earlier than neural crest cell migration [28]. Therefore, dysgenesis in eyes with lens anomalies might occur at an earlier time, resulting in larger extents of anomalies and worse visual function.

Vascularization density was associated with UBM classifications (*P* = 0.014) and anterior segment developmental abnormality (*P* = 0.047). In addition, eyes with more severe intraocular lesions (*P* = 0.001 for UBM classifications, *P* = 0.008 for dysgenesis, and *P* = 0.003 for angle closure extent) appeared to have a larger range of vascularization. One possible reason was that vascularization in CCO eyes was caused mainly by combined dysgenesis of corneal limbal stem cells. All eyes with dysgenesis in our classification had concomitant iris hypoplasia. Pax6, the major gene controlling ophthalmology development, was found to be responsible for limbal cell deficiency in congenital aniridia eyes [30]. In addition, anterior synechia was observed in eyes with mutations in PITX2, FOXC1, and FOXC2, which are required for anterior segment development and angiogenic privilege [17, 31, 32]. Therefore, abnormal findings in superficial and intraocular examinations may be a corresponding expression of the same pathogen.

Moreover, eyes with bilateral CCO seemed to have more severe intraocular anomalies (*P* = 0.019 for UBM classifications, *P* = 0.012 for dysgenesis, and *P* = 0.009 for angle closure). These results support the hypothesis that abnormalities that are present in the process of eye development play a crucial role in the intraocular anomalies of CCO. Compared to causes of unilateral CCO such as trauma and dermoid, abnormal development are likely to cause bilateral CCO, as well as abnormalities in other system. Besides, our data were from a tertiary hospital, and most of the patients had a strong willingness for surgery, so the patient population may have complex bilateral CCO. However, the severity of vascularization was not related to bilateral keratopathy, which indicated that the severity of ocular surface lesions was more susceptible to the severity of intraocular anomalies.

Using UBM examination, we classified CCO eyes into four groups based on intraocular structural anomalies. Similarly, Chen et al. classified 40 eyes with Peters anomaly and Rieger’s anomaly eyes into four groups based on UBM findings, in which corneal echo, iris anomaly, lens anomaly, pupil heteromorphism, and depth of the anterior chamber were considered [33]. Waring’s classification of anterior chamber cleavage syndrome was referred to in Chen’s study, which aimed to ensure balloon UBM’s applicability for children and consequently found it suitable for PA and RA diagnosis [34]. However, neither classification system took ocular surface anomalies into consideration, and the sample sizes were relatively small. In addition, Chen’s classification system focused on specific clinical diagnoses, which was considered confusing, as mentioned earlier. Our study proposed a structural classification system for CCO eyes based on a large sample regardless of their clinical diagnosis and found it to be correlated with the severity of ocular surface anomalies, which might be valuable for surgical prognosis judgement for CCO eyes and intraocular structure evaluation for patients who cannot cooperate with UBM examinations.

However, potential limitations should be noted. First, our classification system relies on the use of UBM, which is not available in some ophthalmology departments. In addition, the patients’ cooperation were required to observe the anterior chamber angles intensively during UBM examinations, but this was impossible for children under anesthesia [35]. Second, the number of cases was comparatively small due to the low incidence of CCO [1, 2]. Third, there has been little discussion about the gradings of CCO and vascularization, so references of our gradings were those of other primary diseases, which weakened the representativeness of the severity of CCO disease. Therefore, further work is needed to validate our classification system and study the correlation between these structural anomalies and surgical prognosis in a larger population of CCO patients.

## Conclusions

In conclusion, we came up with an anterior segment structural classification method based on UBM findings in large sample, which was highly correlated with severity of ocular surface anomalies. Our classification system may be valuable for prognosis judgment for CCO eyes and has significance for reference for surgical procedure design of paediatric patients who are incapable of cooperating with UBM examinations.

## Data Availability

The data that support the findings of this study are available on request from the corresponding author. The data are not publicly available due to privacy or ethical restrictions.

## Abbreviations

CCO: Congenital corneal opacity
UBM: Ultrasound biomicroscopy
